# Drug target of natural products and COVID-19: how far has science progressed?

**DOI:** 10.1097/MS9.0000000000000703

**Published:** 2023-04-19

**Authors:** Kannan Raman, Kalirajan Rajagopal, B. Ramesh, P. Kumar Nallasivan, M. K. Mohan M. Raja, Srikanth Jupudi, Gowramma Byran, Sharuk L. Khan, Talha Bin Emran

**Affiliations:** a Department of Pharmaceutical Chemistry, JSS College of Pharmacy (JSS Academy of Higher Education & Research), Ooty, The Nilgiris, Tamil Nadu India; b Kakatiya Government College, Hanumakonda, Telangana India; c Department of Pharmaceutical Chemistry, Faculty of Pharmacy, Karpagam Academy of Higher Education, Coimbatore India; d Parul Institute of Pharmacy and Research, Parul University, Vadodara, Gujarat India; e Department of Pharmaceutical Chemistry, N.B.S. Institute of Pharmacy, Ausa, Maharashtra, India; f Department of Pharmacy, BGC Trust University Bangladesh, Chittagong, Bangladesh; g Department of Pharmacy, Faculty of Allied Health Sciences, Daffodil International University, Dhaka, Bangladesh

**Keywords:** ACE2, COVID-19, *in silico*, natural product, SARS-CoV-2, spike protein

## Abstract

The new coronavirus [severe acute respiratory syndrome coronavirus 2 (SARS-CoV-2)] that caused a viral disease with a high risk of mortality (coronavirus disease 2019) was found toward the end of 2019. This was a significant acute respiratory syndrome. In a brief period, this virus spread throughout the entire planet, causing tremendous loss of life and economic damage. The process of developing new treatments takes time, and there are presently no recognized specific treatments to treat this infection. The most promising participants, who subsequently developed into prospective leads, were dropped from the clinical research in their latter phases. Medication that has previously acquired permission may only be repurposed for use for various medical reasons following a thorough investigation for safety and effectiveness. Because there are now no effective treatments available, natural products are being used haphazardly as antiviral medications and immunity boosters. The fundamental statement that most natural compounds have powerful antiviral action does not apply to SARS-CoV-2. Middle East respiratory syndrome coronavirus and severe acute respiratory syndrome coronavirus infections are inhibited by natural treatments. According to an *in silico* study, the virus’ nonstructural proteins, including PLpro, Mpro, and RdRp, as well as structural proteins like the spike (S) protein, have been shown to have a strong affinity for several natural products and to be inhibited by them. The virus also suggests that it is a valid candidate for therapeutic research since it utilizes the intracellular angiotensin-converting enzyme 2 receptor of the host cell. In this study, interesting targets for SARS-CoV-2 medication development are explored, as well as the antiviral properties of some well-known natural compounds.

## Introduction

Highlights

- Severe acute respiratory syndrome coronavirus 2 produced a viral illness with a high risk of death.
- Natural products are being used haphazardly as antiviral medications.
- PLpro, Mpro, RdRp, and S protein have been shown to have a strong affinity for natural products.
- In this study, targets for severe acute respiratory syndrome coronavirus 2 medication development are explored.

Many ailments affecting both humans and animals are caused by viruses. According to a recent study, type I diabetes, hepatoma, and brain disorder disease have all been associated with viruses^1–3^. The likelihood of epidemic outbreaks rises with increased urbanization, international travel, and immigration such viral epidemics occurring could exhibit a major threat to the health of individuals as the majority of viral infections do not have effective vaccines or antiviral therapeutics. In a positive sense, the SS-RNA genome of coronaviruses (CoVs) ranges in size from 26.2 to 31.7 kb and is encased in an enclosed form^4^. The shape might be pleomorphic or spherical, and it has several club-shaped glycoprotein projections on its surface, each of which is between 80 and 120 nm in size. CoV’s Ribonucleic acid (RNA) genome is exceptionally large in comparison to those of other RNA viruses^5^. Open reading frames (ORFs) in the range of 6–10 are present in the CoV genomes. The recurring recombination processes that can occur due to the genetic makeup of CoV can lead to noval strains emerging with altered pathogenicity due to mutation^6^. Seven CoV strains, the infection is brought on by a variety of CoV, particularly when the respiratory tract is affected, such as 229E, NL63, OC43, HKU1, Middle East respiratory syndrome (MERS)-CoV, severe acute respiratory syndrome (SARS)-CoV, and 2019-novel coronavirus (nCoV). These symptoms include the common cold, pneumonia, bronchiolitis, rhinitis^7^, 2019-nCoV, SARS-CoV, and MERS-CoV three of these seven strains that were very pathogenic and caused an endemic of severe CoV illness^8^. No one knows where SARS-CoV comes from, despite predictions that it may spread to bats and then to Himalayan palm civets. Additionally, a Middle Eastern zoonotic disease, MERS-CoV is spread through camels^9^. The second MERS-CoV outbreak first appeared in Saudi Arabia in 2012, while the SARS-CoV pandemic first appeared in the Chinese region of Guangdong in 2003. CoV was previously thought to only produce lesser sickness, but these two outbreaks demonstrated their capacity to adapt to shifting environmental conditions, which is why they are categorized as emerging viruses. To find potential pharmacological targets, it is critical to understand the structure, metabolic processes, and path physiology of CoV-associated diseases^10^.

Four of the major structural proteins of CoV are the beta-CoVs and nucleocapsid protein (N), membrane protein (M), trimeric, envelop protein (E), and spike protein (S). Other viruses include the hemagglutinin esterase (HE) glycoprotein^6^. The seven conserved genes in the RNA genome of CoV are N in the 5′ to 3′ direction, ORF1a, OEF3, E, M, S, and ORF1b. The ORF1a/b gene pair, which generates the two polyprotein viral replicase proteins, covers two-thirds of the RNA genome (PP1ab and PP1a)^11^. Sixteen functional nonstructural proteins (NSPs) are ultimately derived from these two protein phosphates. The creation of the replica’s transcriptase complex is one of the viral processes in which these NSPs participate. The virus’s leftover genome encodes the mRNA that creates structural proteins such as the nucleocapsid, spike, membrane, and envelopes in addition to various auxiliary proteins. The HE protein, which is exclusively generated by a few CoV strains, is an essential envelop-associated protein^12^.

**Figure 1:**
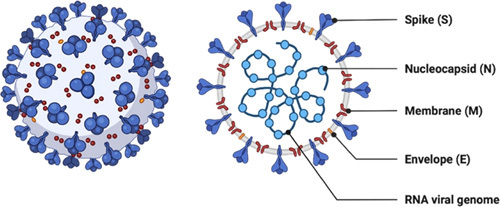
Structure of human coronavirus.

Early research found that the 2019-nCoV substantially grouped with the bat SARS-like CoV sequence obtained in 2015, while structural analysis exposed mutations in the spike glycoprotein and nucleocapsid protein. So, it is evident that the new 2019-nCoV is different from the SARS virus, which was most likely spread by bats before undergoing a mutation that allowed it to infect people. The CoV Study Group designated this virus as severe acute respiratory SARS-CoV-2 based on the phylogenetic tree, taxonomy, and accepted practice. The current CoV-associated acute respiratory illness epidemic was given the name COVID-19^13,14^. The nucleocapsid protein and an additional envelope surround the RNA genome of CoV (Fig. 1).

**Figure 2:**
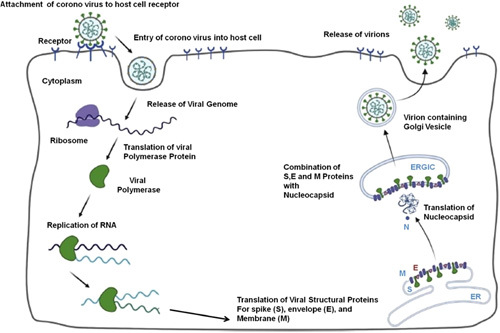
Lifecycle of COVID-19 virus inside the host cell.

For the creation of drugs against SARS-CoV-2 infection, it is critical to comprehend the CoV life cycle and pathophysiology. The flow cycle of CoV inside the host cell is depicted in Figure 2. Virion attachment to cellular receptors is the first step in the development of SARS-CoV-2 infections. After a successful attachment, the virus enters the host cell and replicates freely in the cytoplasm. The protein called viral polymerase is responsible for translating viral RNA. After replication and the generation of subgenomic RNA, the viral structural proteins, such as envelope (E), membrane (M), and spike (S) are translated and incorporated into the endoplasmic reticulum. These proteins enter the membranes of the Golgi intermediate compartment through the secretory pathway where they bind to the nucleocapsid (N) protein. The mature virion is formed by the virus once it enters the Golgi vesicle. To release the replicated viral cells into the host body, the membrane of the host cell and the vesicle holding the virion fuse.

## Research opinion on SARS-CoV-2 and antiviral drug development

The detection mechanism of RNA and nucleotide inhibitors by RNA-dependent RNA polymerase is discussed in the review^15^ along with its relevance to drug development. The article highlighted experiences and developments with previous SARS- and MERS-CoVs, which combined might allow attempts to stop this new viral infection. It also emphasized current developments in developing vaccines and medications to combat COVID-19. Major acute respiratory syndrome the CoV illness of 2019 is caused by the SARS-CoV-2 virus, which has positive single-stranded RNA in its genetic makeup^15^. The scientific community is informed by work like this, which encourages further study and the use of natural ingredients in the hunt for novel COVID-19 antiviral. The development of new medications to completely limit the illness is challenging, making cost-effective drug repurposing a far more practical strategy^16^. As a consequence, guaifenesin’s action causes a cough to be more productive. This is also anticipated to occur if provided to COVID-19 patients since it may prevent the E-pentameric protein’s structure. A review described the different computational strategies used to produce medications via drug repositioning or repurposing against COVID-19 and discussed their benefits and drawbacks^17^. Since there is now no effective treatment or vaccine, the recent appearance of a new coronavirus (SARS-CoV-2) has posed a danger to human civilization. Novel medication candidates are in high demand due to rising morbidity and mortality^18^. Six components have been identified as possible therapeutic candidates that may prevent the SARS-CoV-2 spike protein from interacting with the human receptor angiotensin-converting enzyme 2 (ACE2) protein as a result of molecular docking^19^. This study plant metabolites that have the therapeutic potential to cure a variety of viral infections. In this research, remedies from plant families were sought, as well as candidates for susceptible viruses, antiviral tests, and the mechanism of therapeutic action. This effort led to the gathering of a vast array of natural medicines that might be recommended for the treatment of COVID-19.

## Clinical data on potential COVID-19 FDA-approved therapeutics

The unique SARS-CoV-2 virus gave rise to the viral illness known as COVID-19, which had consequences for the economy, human health, society, and other factors. The absence of a line of therapy that the FDA has approved puts a lot of pressure on collaborative in medical and scientific fields to come up with novel therapeutics^20^. The technique of developing a product is made more difficult by the mechanism, efficacy, and toxicity profiling of a substance at different stages. In 1906, to ensure the effectiveness and security of possible pharmaceuticals and medical equipment, Congress enacted the Food and Drugs Act^13^. The typical procedure for creating new medications includes the following steps: research and development, postmarket surveillance, clinical trials, and preclinical research^21^.

Medication development in the USA takes about 10 to 12 years, and only 9.6 to 13.8 percent of all drug proposals are approved by the FDA^22,23^. Target selection and validation, lead identification, and optimization are all steps in the drug discovery process^24,25^. The main areas of focus in the preclinical phase include active pharmaceutical ingredients (API) production, analysis techniques for medication creation, and formulation of ADMET criteria, and good manufacturing practices^19^. After the aforementioned procedures have been completed successfully, API will be used in the first stage of clinical trials^20^. This stage typically involves 20–100 human participants, and the primary goal is to measure the dose and safety profile. The number of human candidates increases in phase II clinical studies to examine the efficacy and side effects of the medicine. Phase III aims to evaluate the participant drug’s efficacy in comparison to currently available therapies and has more than 3000 human applicants. After the drug candidates have completed the aforementioned stages, the scrutinizer’s analysis of a new drug application takes around 10 months^26^. Phase IV is regarded as the postmarketing phase of drug safety monitoring^27^.

**Figure 3:**
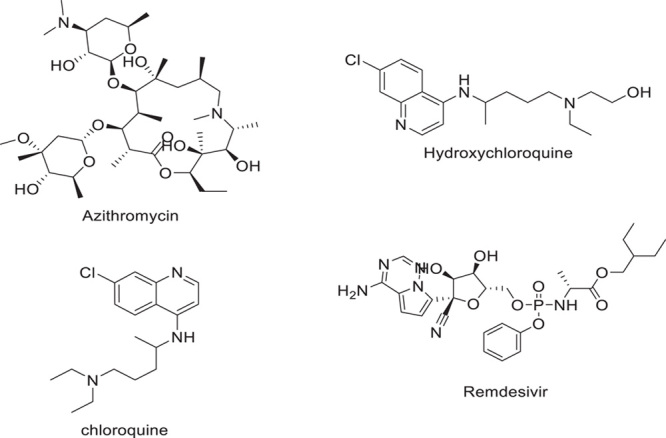
FDA-approved drug for SARS-CoV-2.

To create the COVID-19 therapies, researchers analyzed the data already available on the replication and life cycle of SARS-CoV-2^28^. The quickest way to develop an efficient COVID-19 treatment is through the repurposing of FDA-approved medications. Hydroxychloroquine, a drug used to prevent and treat autoimmune diseases and malaria, was initially tested as a therapy for SARS-CoV-2. The other common antiviral drug, redeliver, has been effective in treating previous human CoV infections. Remdesivir is a comparably effective RNA polymerase inhibitor, and adenosine GS-441524 is efficient against RNA-dependent viruses. Both *in vitro* and *in vivo* studies have focused on Remdesivir’s potential as a treatment for SARS-CoV and MERS-CoV^28,29^. Remdesivir was found to be active against multiple CoV in humans, including MERS-CoV, SARS-CoV, and bat CoV, according to a study published in 2017^30^. In cell culture assays, the 50% effective concentration of redeliver against SARS-CoV and 50% effective concentration of 0.074 M against MERS-CoV were observed. Within 4 days of infection, the viral load in the lungs of an animal model was reduced by greater than 2 orders of magnitude when treated with remdesivir[60]. In SARS-CoV, remdesivir is also used prophylactically to enhance breathing and alleviate clinical symptoms. Both *in vitro* and *in vivo* research have shown that Remdesivir is effective against SARS-CoV. Human trials and preclinical research had already established remdesivir’s safety and efficacy in treating other RNA viruses before the development of COVID-19^31^. Grein^32^ conducted pioneering research with remdesivir to treat COVID-19. The findings were reported in the NEJM. Fifty-three people were randomly selected to receive intravenous Remdesivir for 10 days. According to the authors’ findings, 25 patients had successfully recovered after 18 days, 17 had been extubated from artificial breathing, and 7 had passed away. Chloroquine has been provided FDA approval for the treatment of rheumatoid arthritis and systemic lupus erythematosus. Chloroquine reduced viral replication and endosomal/lysosomal antigen processing in early investigations^33^. Chloroquine improved the survival rates of newborn mice infected with the human coronavirus in animal models^34^. Due to ongoing *in vitro* investigations, chloroquine and hydroxychloroquine were the only effective treatments for SARS-CoV-2 in December 2019 in China^35^. According to research by Yao *et al*., hydroxychloroquine had a superior safety profile than chloroquine for treating COVID-19. As a result, this medication was a desirable choice for the treatment of COVID-19 in particular because it was FDA-approved for a variety of applications and had up-to-date documentation^36^. Azithromycin plus antimalarial medications were considered to be an effective alternate treatment for SARS-CoV-2.66. There were no fatalities when azithromycin was administered along with chloroquine and hydroxychloroquine to SARS-CoV-2 patients^37,38^. The dilemma of whether the medications can be administered separately or in combination arose from the cardiotoxicity that the antimalarial treatments alone or in combination with azithromycin induce, which enhances the adverse responses. The QT intervals were lengthened in the described cohort research when azithromycin was combined with chloroquine or hydroxychloroquine^39^. Azithromycin did not extend QT intervals clinically; however, the combined treatment did so noticeably^40^. According to these investigations, the combination of azithromycin and chloroquine/hydroxychloroquine for SARS-CoV-2 treatment may be avoided (Fig. 3).

## Currently used COVID-19 management strategies

Clinicians are pursuing Convalescent Plasma Therapy (CPT) as an immunotherapeutic alternative to antiviral medication therapy^41–44^. While antiviral medications are undergoing clinical testing, CPT is emerging as a potential COVID-19 therapeutic option. The infected patients receive plasma obtained from recovered participants with this adaptive immunotherapy. A high titer of neutralizing antibodies, which can have an antiviral impact, is present in the plasma^45^. Studies have demonstrated that CPT is effective in opposition to COVID-19 and that there have been no serious adverse responses to this treatment. A similar element of this therapy was revealed in additional research as well. A 200 ml dosage of CP with a neutralizing antibody titer greater than 1 : 640 was administered to 10 critically sick patients in Chinese pilot research (ChiCTR2000030046). In seven patients out of 10, there was a notable clinical and radiological improvement as well as a decreased viral load^46^. An alternative study showed that after 12 days of CPT, a patient’s Sequential Organ Failure Assessment score dropped from 2 to 10 before treatment to 1–4^47^. This study also showed that the Virus-specific IgG and IgM antibody titer value was dramatically greater in five critically sick patients and that the development of the viral infection was slowed in all of the patients. A study revealed a potential disadvantage of this course of treatment: 3 months after starting CPT in MERS-CoV-infected individuals, the antibody titer rapidly decreased. Since transfusion is a component of the therapy, CPT also has the risk of transfusion-transmitted infection^48^. Antihuman leukocyte antigen antibodies are the source of TRALI, and a similar case was noted for CPT during an Ebola virus outbreak^49^. Before beginning CPT for COVID-19, it is advised to screen for antihuman leukocyte antigen antibodies^45^. Finally, because the plasma is human-derived, it must be obtained and used under the strictest ethical standards for CPT to be successful^41^.

## The justification for natural products as a possible line of COVID-19 treatment

**Figure 4:**
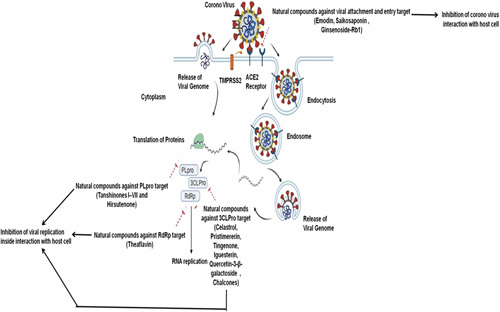
Natural compounds and their possible inhibition mechanisms against COVID-19.

The pandemic scenario is somewhat improved by the aforementioned medications and the CPT. Nevertheless, it is challenging to apply CPT globally because of the expensive therapy, restricted availability, unprecedented adverse medication effects, and ethical questions around it. Since most antiviral treatments work by targeting specific viral proteins, the problem may get much worse if the virus were to develop into a drug-resistant mutant^50^. Thus, natural products can be considered in the quest for potential remedies. Herbal treatments and other natural things have been the subject of a great deal of research due to their potential to combat viruses. The method of action of these natural products as well as potential targets such as viral entrance, replication, assembly, release, and interactions particular to the virus and the host has been the subject of extensive research^51^. Some of the main mechanisms in protecting host cells against COVID-19 include inhibition of viral interaction with the host cell and inhibition of viral replication within a host cell. Figure 4 depicts the natural compounds and their action mechanism against COVID-19. To inhibit viral interaction with a host cell, the binding of COVID-19 spike (S) protein with the host cell receptors must be inhibited. Hence, S protein is one of the potential targets for COVID-19 drugs. Natural substances that target S protein include emodin, saikosaponin, and ginsenoside-Rb1 to exhibit anti-COVID activity. The viral genome also serves as the messenger RNA that is used by the host cell to translate into proteins such as papain-like cysteine protease (PLpro), 3CLPro, and RdRp. RdRp, PLpro, and 3CLPro are appealing targets for anti-SARS-CoV-2 medications since RdRp is necessary for viral replication, while PLpro and 3CLpro are necessary for proteolytic action. Inhibiting the 3CLpro activity is how natural compounds like Celastrol, Tingenone, Pristimererin, Quercetin-3-galactoside, Igesterin, and Chalcones combat SARS-CoV. Natural products including Hirusutenone and Tanshinone I inhibit PLpro activity to combat SARS-CoV. Natural substances like Theaflavin concentrates on the inhibition of RdRp. Thus, inhibition of spike, PLpro, 3CLpro, and RdRp targets by natural compounds exerts potential anti-COVID activity. In the sections that follow, we will go through various potential antiviral drugs that could be utilized to treat and prevent COVID-19.

### Polyphenols’ role in battling the SARS/COVID-19 pandemic

**Figure 5:**
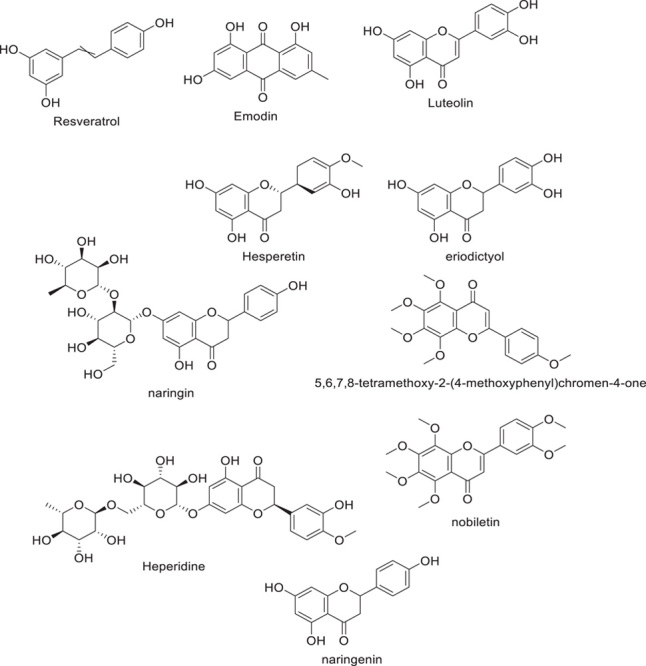
Polyphenol’s role in batting the SARS/COVID-19 pandemic.

Medications that attack the virus itself, and most of the possible treatments for SARS-CoV-2 involve medications that work on the organism and its immune response^46^. The target proteins in SARS-CoV-2 are divided into two groups: NSPs (Mpro, PLpro, and RdRp) and the spike protein. In an *in vitro* investigation, the well-known phytoalexin resveratrol demonstrated strong inhibitory activity against MERS-CoV. Resveratrol may help cells survive longer following viral infection, according to the same study^52^. Anthraquinone polyphenol emodin, isolated from rhubarb roots, has been shown to inhibit the interaction between ACE2 and S protein^48^. Curcumin and its derivatives, as well as the citrus polyphenols hesperetin, hesperidin, and tangerine, bind to the S protein more strongly than the standard chemical nafamostat, according to research using molecular docking^53^. Antiviral remdesivir, which is authenticated by the FDA for COVID-19 treatment was found to attach to S protein with significantly less energy than naringenin^54,55^. Luteolin and tetra-O-galloyl-d-glucose (TGG) were observed to attach to the protein on the surface of the CoV and prevent the entry of the virus into host cells (Fig. 5)^56^.

**Figure 6:**
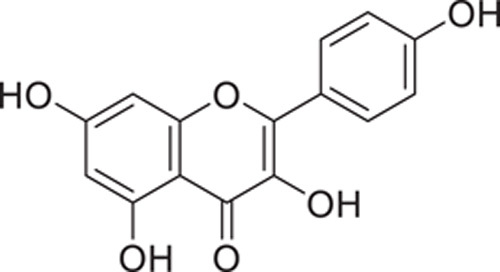
Kaempferol.

The transmembrane metallocarboxypeptidase ACE2 serves as the SARS-binding CoV-2’s site^57^. As a result, this receptor is a promising new avenue for the creation of antiviral medications. Out of 77 possibilities, the flavanone eriodictyol from *Eriodictyon californicum* had the strongest affinity for ACE2^58^. More *in vitro* and *in vivo* investigations are needed to determine the candidates’ real effects on the pandemic, even though *in silico* studies can help discover interesting candidates. According to a study, significantly serious lung damage and SARS-CoV infection occurred in mice with inactive or managed to knock ACE2 versus wild-type controls. Recombinant ACE2 was administered, and the symptoms subsided^59^. An *in vitro* assay demonstrated that injection of soluble ACE2 blocked the entry of SARS-CoV and SARS-CoV-2, indicating transgenic ACE2 could serve as a decoy target for the S protein of viruses^60,61^. Since ACE2 is crucial to human physiology, it should only be targeted for antiviral medication development after careful consideration of the dangers. A class of drugs called protease inhibitors has been widely utilized to treat viruses like HIV, MERS-CoV, and SARS-CoV^43,62^ by performing proteolytic processing on the polyprotein, 3CLpro (Mpro) and PLpro separate the proteins both structural and nonstructural required for the life cycle of CoV^63^. The SARS-CoV proteases are effectively inhibited by natural products such as coumarins100, flavonoids, terpenoids^64,65^, and diarylheptanoids. Gallocatechin gallate (IC50=47 µM), quercetin (IC50=73 µM), and epigallocatechin gallate (IC50=73 µM) are all significant inhibitors of Mpro in SARS-CoV-2, according to *in vitro* and *in silico* investigations^66,67^. The SARS-CoV-2 Mpro and PLpro were synergistically suppressed *in vitro* by flavonoids like kaempferol and isoliquiritigenin. Through the use of molecular docking analysis, to locate potent inhibitors of SARS-CoV-2 Mpro, Gentile *et al*.; examined a collection of marine natural products (MNP Library). The most remarkable inhibitors were found in Ecklonia cava (8,8′-bieckol, 6,6′-bieckol, and Dieckol) and *Sargassum spinuligerum* (1,3,5-trihydroxybenzene, heptafuhalol A, phlorethopentafuhalol A, phlorethopentafuhalol B, phlorethopentafuhalol A Herbacetin, rhoifolin, as well as pectolinarin are only some of the flavonoids found in traditional Chinese remedies that have been shown to suppress the Mpro of SARS-CoV (Fig. 6). Herbacetin, isobavachalcone, and helichrysetin are examples of flavonoids that Jo *et al*. discovered have an inhibitory effect on MERS-CoV Mpro. To determine how well over 200 plant extracts inhibited SARS-CoV, Wen *et al*. Vero E6 cell lines were used to examine the cytopathogenic effects of SARS-CoV, and it was found that indicated 25–200 g ml-1 quantities of extracts from the herbs *Gentianae radix, Dioscoreae rhizoma, Cassiae semen, Loranthi ramus*, and *Rhizoma cibotii* may have an inhibiting effect on SARS-CoV. Three potential leads that limit SARS-CoV-2 infection by blocking the target protein TMPRSS2 of the host were found through a recent *in silico* examination of naturally occurring substances. Glucogallin, mangiferin, and phlorizin were all identified in the same study, and their use in limiting the Mopar. 108 viruses’ ability to replicate within hosts was explored^68–71^.

### Alkaloids’ part in the combat against the COVID-19 pandemic

**Figure 7:**
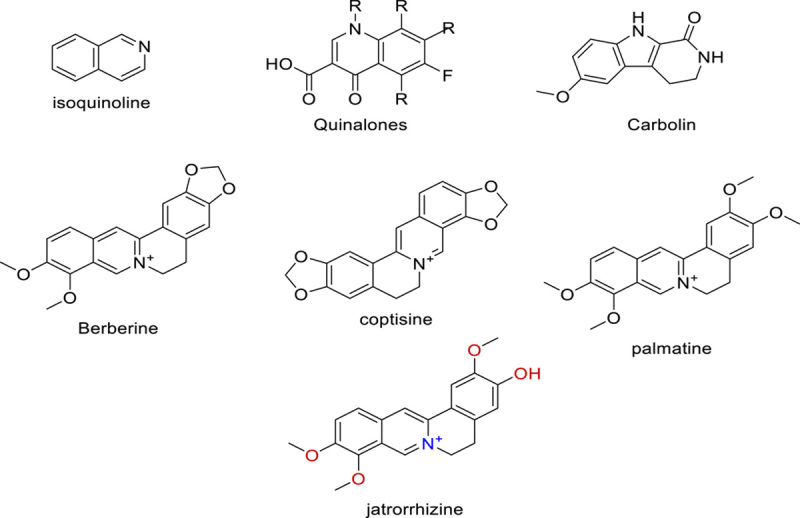
Isoquinoline alkaloids.

**Figure 8:**
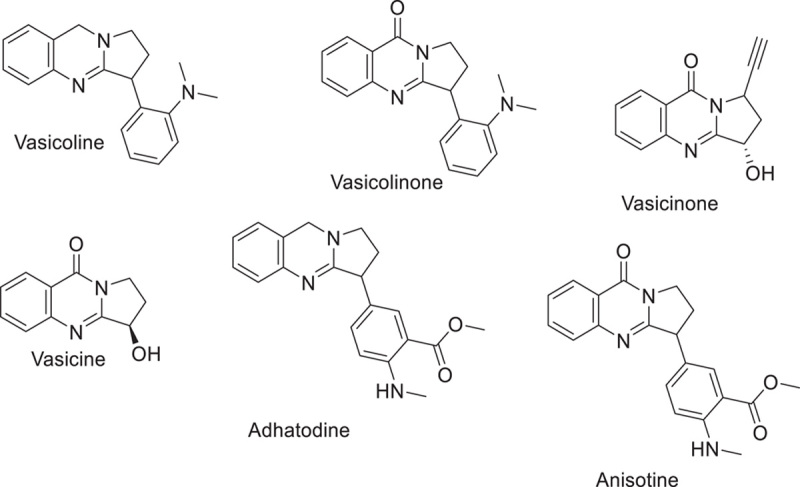
Alkaloids from of *J. adhatoda*.

The chemical structure of alkaloids has a minimum of one nitrogen atom. Alkaloids can be obtained by a wide range of organisms, including fungi (such as Psilocybin’s psilocybin), animals (such as toad skin’s bufotenin), and terrestrial plants. The marine creatures additionally create alkaloids in addition to these. A virus’s genetic makeup can be either DNA or RNA. Several alkaloids target RNA or DNA. DNA intercalating agents are often lipophilic, planar aromatic compounds, and stack between base pairs of DNA or RNA. Strong antiviral activity and intercalating capabilities are exhibited by the alkaloids isoquinoline, quinolone, and carboline. Several isoquinoline alkaloids were found to inhibit SARS-CoV replication. These included berberine, berberine, coptisine, berberrubine, jatrorrhizine, doctrine, palmatine, fangchinoline, tetrandrine, and cepharanthine. Chloroquine (alkaloid quinine derivative) lends credence to the belief that intercalators are potent antiviral medicines against SARS-CoV-2^72,73^. Before designating the intercalating alkaloids as supplementary treatment candidates, a more thorough investigation should be carried out. Schizanthine Z, a tropane alkaloid isolated from the Schizanthus porridges plant, has been shown in molecular docking research to bind strongly to the viral PLpro and to inhibit the protease^73–75^. Alkaloids 10-hydroxyusambarensine (from *Strychnos usambarensis*’s roots)^73^, cryptoquindoline (from *Cryptolepis sanguinolenta*)^74^, and cryptospirolepine (from *Cryptolepis sanguinolenta*)^75^ were all reported to have high affinities for linking to Mpro of SARS-CoV-2 in computer simulations. 10-hydroxyusambarensine and cryptospirolepine were found to have the greatest linkage with the 3CLpro in coronavirus, respectively, in the same study^75^. Another *in silico* study showed that the alkaloids obtained from *Cryptolepis sanguinolenta*, notably cryptoquindoline, biscryptolepine, cryptomisrine, and cryptospirolepine, attach to the RdRp, illustrating that they might be remarkable inhibitors of RdRp. Molecular dynamics studies also demonstrated that alkaloids from the leaves of *J. adhatoda*l, including vasicoline, adhatodine, vaccine, and anisotine and significantly inhibited the SARS-CoV-2 Mpro, making them particularly promising for the protease inhibitor family of drugs (Figs. 7 and 8)^76,77^.

### Glycosides and terpenoids’ role in battling the COVID-19 pandemic

Terpenoids, also known as isoprenoids, are a broad class of naturally occurring substances that are formed from isoprene (5-carbon molecules). Terpenes are created when the isoprene monomers polymerize. Terpenoids provide a wide range of therapeutic benefits, including antiviral activity, antibacterial activity, antioxidant activity, etc. Wen *et al*. looked at the ability to suppress SARS-CoV in more than 200 found in nature terpenoids and lignoids. Examination of the cytopathogenic activity of the compounds in response to virus infection on VERO E6 cell lines revealed that 7-hydroxydeoxycryptojaponol, ferruginol, betulonic acid, and 3-,12-diacetoxyabieta-6,8,11,13-tetraene were the most effective inhibitory compounds. The SARS-CoV Mpro was inhibited by betulinic acid and saving, according to the same study. Terpenoids such as salvinorin A from *Salvia divinorum*, thymoquinone from *Nigella sativa*, bilobalide from *Ginkgo biloba*, menthol from Mentha, citral from *Backhousia citriodora*, noscapine from the Papaveraceae, and ginkgolide A from *Ginkgo biloba* have recently been discovered to have SARS-CoV Mpro inhibitory action, according to Several quinone methide triterpenes isolated from *Tripterygium regelii*, including celastrol, pristimerin, tingenone, and iguesterin, were found to have strong inhibitory activity against SARS-CoV Mpro. Tanshinone, a compound discovered from *S. miltiorrhiza* that contains the abietane diterpene moiety, exhibited selective inhibition against SARS-CoV Mpro and PLpro^78–83^.

**Figure 9:**
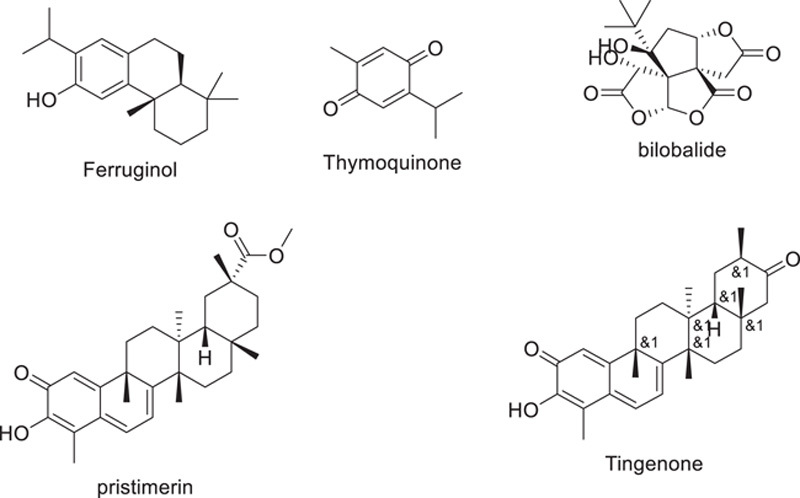
Glycosides and terpenoids’ role in battling the COVID-19 pandemic.

During prior pandemics, such as MERS-CoV and SARS-CoV, cardiac glycosides like digitoxin and digoxin were examined^84,85^. According to the investigations, these cardiac glycosides can deplete the host’s intracellular potassium, which prevents the Na^+^-K^+^-ATPase pump from signaling the virus to internalize through the membrane. Because of this, the highly contagious life cycle within the host was broken. According to a 2005 study by Yang *et al*., cardiac glycosides suppress many cytokines, including NF-kB, TNF, and others^86^. Furthermore, coronavirus infection of the target organ was associated with a cytokine storm^87^. Therefore, following a thorough clinical evaluation, the use of cardiac glycosides as a therapy for SARS-CoV-2 may be considered. Using a molecular docking method, the effective barriers of RdRp and Mpro in COVID-19 were observed to be the flavonoid glycosides namely nicotiflorin and rutin from *Dysphania ambrosioides*, and their respective sulphate, and glucuronide derivatives (Fig. 9)^88^.

### Marine-derived natural products are a novel source act against SARS/COVID-19

**Figure 10:**
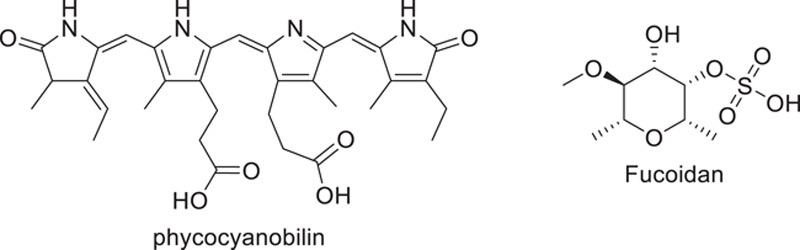
Marine source against SARS-CoV-2.

There has been some recent interest in the therapeutic potential of marine-derived natural compounds. Marine microalgae, which belong to Phaeophyta and Rhodophyta contain a variety of bioactive substances, including the antioxidants phycocyanin and lutein, as well as polysaccharides, vitamins, and phenolics, all of which have been shown to have antimicrobial, anticancer, antiinflammatory, and other important pharmacological effects^88^. Phycocyanobilins come under the tetrapyrrole chromophores class and they are observed in some marine cyanobacteria; in 2010, Hirata *et al*. studied their potential antiviral and antioxidant effects^89^. *In silico* molecular docking studies found that phycocyanobilins strongly bind to SARS-CoV-2 Mpro and RdRp^89^. A group of substances known as lectins has an affinity for carbs. Studies on the lectin griffithsin, which is generated from red algae, have demonstrated that it has antiviral action against hepatitis C and HIV-1. Griffinsin inhibited MERS-CoV, millet *et al*. found in their most recent *in vitro* investigation. Brown microalgae *Sargassum henslowianum*’s sulfated polysaccharides, often known as fucoidans, have been demonstrated to have a potent inhibitory effect on HSV *in vitro* studies^90,91^. According to research fucoidans isolated from the macroalgae *Saccharina japonica* exhibited remarkable anti-COVID activity. The RPI-27 fucoidan, one of the two fucoidans used in their investigation, exhibited greater anti-COVID efficacy than that of redeliver. They determined that fucoidans in conjunction with other antiviral medicines can produce significant anti-COVID action. The ethyl ester obtained from the marine sponge Axinella cf. corrugate, achieved a better linkage with the protease in coronavirus, making it a promising candidate for use as an antidrug against COVID-19. Carrageenan is a class of sulfated polysaccharides found in marine organisms that are thought to hinder the replication of several different viruses. Because they stop the virus from adhering to and entering the host cell, they are effective. It was hypothesized that these chemicals can be utilized to coat sanitary objects for preventing infection from COVID-19. Recently, in silico studies are utilized to discover potential lead molecules for COVID-19 pandemic medication development. Using a molecular dynamic approach, five marine-derived (1-hexadecoxypropane-1,2-diol, fostularin 3, 15alpha-methoxypuupehenol, palmitoleic acid, and puupehedione) barriers of Mpro in COVID-19 with the potential to block viral process within the host (Fig. 10). It is still difficult to obtain significant natural compounds from marine sources because of the rarity, scarcity, and difficulty of collecting marine environment. The hunt for strong natural substances from marine sources that can demonstrate a variety of actions has been ongoing and producing promising results over the past few decades. Therefore, we can generalize these results to discover a potential treatment using the sea species that exist naturally.

## Application of phytochemical in countering COVID-19

Because COVID-19-targeted conventional medicines like vaccinations, antibiotics, etc. are not now available, people are turning to broad-spectrum antibiotics and tried-and-true antiviral medications instead. Here, the CoV can be effectively fought using extracts from naturally occurring products that are a rich source of active compounds^92^. SARS-CoV replication has been seen to be inhibited by extracts from numerous traditional Chinese medicinal herbs. In a study, over 200 extracts were looked at, and it was determined how effective they were at fighting the SARS-CoV^5^. The replication of SARS-CoV 3CLpro was successfully inhibited by bioflavonoids produced from *Torreya nucifera* ^93^. At a concentration of 100 g/l, the ethanolic extract made from this plant’s leaves exhibited 62 percent inhibitory activity. The fluorescence resonance energy transfer technique was used to demonstrate the possible inhibitory effects of eight diterpenoids and four bioflavonoid. The molecular docking study provided support for the experimental findings of the enzymatic assays. The antiviral activity of *Houttuynia cordata* Thunb extracts including quercitrin, quercetin, and cyan-serine was examined *in vitro* studies on mice infected with CoV and dengue virus^94^. The flavonoid was examined for oral dose toxicity in rats as well as their capacity to neutralize the mouse coronavirus and dengue virus. For the coronavirus and dengue virus, the extracts’ IC50s were 0.98 mg/ml and 7.50 mg/ml, respectively. Mice were given oral dosages of up to 2000 mg/kg without experiencing any acute toxicity. In addition, a synergistic interaction between quercetin and quercitrin’s antiviral activity was discovered. Flavonoids were studied for their possible action against the MERS-CoV-3 coronavirus^68^. Helichristetine, herbaceous, quercetin 3-d-glucoside, and isobavachalcone were identified as compounds that inhibit the enzymatic activity of the MERS-CoV-3 coronavirus. Inhibitors of MERS-CoV 3CLpro have included flavonoids with hydrophobic or carbohydrate groups. Flavonoids were tested on SARS-CoV-infected *Pichia pastoris* by Nguyen *et al*.^63^. The antiviral activity of certain quercetin derivatives against the hepatitis C virus and SARS-associated coronavirus has been investigated^95^. The structure-activity relationships of quercetin-3-galactoside and its derivatives were analyzed to the SARS-CoV 3CLpro^96^. The quercetin-3-galactoside shows potential as an anti-SARS medicine and has helped to get insight into the inhibition mechanism against the target enzyme. Sambucus Formosana Nakai extract, containing chlorogenic acid, coffee acid, and gallic acid as active phenolic acid components, showed excellent anti-HCoV-NL63 potential^97^. It has been demonstrated that the polyphenolic elements in green tea have an antiviral impact^95^. *Toona sinensis* Roem’s leaf extracts had SARS-CoV inhibitory activity^96^. Preparations using either *Galla chinensis* or *Veronica linrrifolia* contain flavonoids that have been shown to bind to the spiky proteins on the surface of the SARS virus and block the virus’s ability to enter cells^97^. The molecular docking study provided evidence to validate the experimental studies. The phenolic compounds found in root extracts of *Isatis indigotica* are effective against SARS-CoV 3CLpro.

## Conclusions and future insights

COVID-19, which has infected over 1 000 000 individuals and continues to do so daily, has emerged as a top target for quick treatment development. The discovery of a new CoV mutation in the UK, identified as VUI-202012/01, sent shockwaves through the world. This unique variety contained 17 alterations; the most significant among these mutations was the N501Y mutation in the S protein, which may increase its contagiousness. The quest for an effective vaccination is underway at research centers around the globe, but developing one takes time. There are supposedly three vaccine candidates in development, but only one made by Pfizer has received WHO EUL/PQ approval. The Moderna, Pfizer, and AstraZeneca vaccines are used on a medium scale all over the world. Press conferences are used to release information about the vaccines’ safety and effectiveness. We must search for small compounds that can successfully lessen the lethality of the CoV till then. Synthetic small compounds like hydroxychloroquine and chloroquine have been utilized to treat COVID-19 patients, but because of their side effects and limited availability, this treatment strategy is usually useless. It has been reported that plant extracts and natural compounds possess strong antiviral activities as well as an inhibition effect on viral proteins, which are required for the life cycle of CoV. Our review provides an important update on numerous natural substances that have shown promise as COVID-19 therapies and anti-CoV drugs. As this review demonstrates, many natural compounds have proven impressive antiviral effectiveness against COVID-19 using a variety of target approaches. These substances might be developed further to produce bioactive derivatives and medicinal leads. We have accumulated a large number of effective antiviral medications from the *in silico* data that need extensive *in vitro* and *in vivo* testing. There is still a long way to go before natural substances are used in therapeutic settings, even though they have shown encouraging outcomes in the treatment of COVID-19. The solubility and bioavailability of natural substances, as well as the significant expense and complexity of clinical trials, are some of the main obstacles to turning them into medications against COVID-19. There are not many pharmacokinetic studies on the fascinating natural compounds against COVID-19 in this review. The pharmacokinetic profile of natural substances against COVID-19 will therefore require further investigation in the future. It may eventually be possible to develop new COVID-19 treatment options as a consequence of research on the enhancement of a few of the key natural chemicals using well-established or unproven ways of action. Optimizing lead natural chemicals against COVID-19 may be one of the treatment trajectories for COVID-19 in the future.

## Ethical approval

This case report has been reported in line with the Case Report (CARE) guidelines.

## Consent

Not applicable.

## Source of funding

None.

## Author contributions

K.R. and K.R.: conceptualization, data curation, writing-original draft preparation, writing-reviewing and editing; B.R., P.N., M.K.M.M.R., S.J., G.B., S.L.K.: data curation, writing-original draft preparation, writing-reviewing and editing; T.B.E.: conceptualization, writing-reviewing and editing, visualization.

## Conflicts of interest disclosure

The authors declare that they have no financial conflict of interest with regard to the content of this report.

## Research registration unique identifying number (UIN)

Name of the registry: Not applicable.
Unique Identifying number or registration ID: Not applicable.
Hyperlink to your specific registration (must be publicly accessible and will be checked): Not applicable.

## Guarantor

Talha Bin Emran, Ph.D., Associate Professor, Department of Pharmacy, BGC Trust University Bangladesh, Chittagong 4381, Bangladesh. Tel.: +880 303 356 193, fax: +880 312 550 224, Cell: +880 181 994 2214. https://orcid.org/0000-0003-3188-2272. E-mail: talhabmb@bgctub.ac.bd

## Provenance and peer review

Not commissioned, externally peer-reviewed.

## Trial registry number

Not applicable.

## Declaration of competing interest

We have read and understood the policy on the declaration of interests and have no relevant interests to declare. The responsibility for the content lies with the author and the views stated herein should not be taken to represent those of any organizations or groups with and for which he works.
